# Relationship between morphometric and mechanical properties of superficial lumbosacral soft tissue layers in healthy young adults

**DOI:** 10.3389/fphys.2023.1175035

**Published:** 2023-05-16

**Authors:** Marcin Grześkowiak, Piotr Kocur, Dawid Łochyński

**Affiliations:** ^1^ Department of Cardiological and Rheumatological Rehabilitation, Poznan University of Physical Education, Poznan, Poland; ^2^ Department of Musculoskeletal Physiotherapy, Poznan University of Physical Education, Poznan, Poland; ^3^ Department of Neuromuscular Physiotherapy, Poznan University of Physical Education, Poznan, Poland

**Keywords:** low back, lumbar myofascial, rehabilitative ultrasound imaging (RUSI), plastic surgery, spinal surgery

## Abstract

**Introduction:** It is commonly considered that myotonometry is a non-invasive method capable of quantifying linear elastic and viscoelastic properties of the myofascial tissue through the application of a weak mechanical impulse to the surface of the skin. However, before the impulse can reach the myofascial tissue, it must cross more superficial tissues such as the skin and subcutaneous tissue (ST). All these superficial tissues have different distributions and organizations of structural components. Therefore, the study aimed to examine the potential relationships between the mechanical and morphometric properties of various superficial soft tissues surrounding the lumbar multifidus muscle (LM).

**Methods:** Myotonometric measurements of dynamic stiffness, logarithmic decrement, and creep, and ultrasonographic measurements of thickness and echogenicity of cutaneous, subcutaneous, perimuscular tissue, and LM were obtained from 50 healthy individuals in the resting prone position and during contralateral arm lift.

**Results:** The most important findings were that in both the relaxed and contracted LM state, the dynamic stiffness strongly negatively (*r* = −0.69; *p* < 0.001 in relaxation, *r* = −0.83; *p* < 0.001 in contraction) and creep strongly positively (*r* = 0.79; *p* < 0.001 in relaxation, *r* = 0.85; *p* < 0.001 in contraction) correlated with the thicknesses of the ST. Similar but weaker correlations were noticed between both these measures and the perimuscular tissue thickness. Elasticity was uncorrelated to the thicknesses of the tissues. With LM contraction (change from the relaxed to contracted state), the relative increase in dynamic stiffness was correlated with the relative decrease in dermis (*r* = −0.51; *p* < 0.001) and ST (*r* = −0.47; *p* = 0.001) thickness, and with the relative increase in LM (*r* = 0.36; *p* = 0.010) thickness. Moreover, the relative decrease (thinning) in the ST thickness was correlated with the relative increase in logarithmic decrement (i.e., decrease in soft tissue elasticity, *r* = −0.37, *p* = 0.011). The mechanical properties of the soft tissues were not related to their echogenicity.

**Discussion:** In conclusion, the thicker the subcutaneous and perimuscular layers, the lesser the stiffness and the greater the time-dependent deformation to the external force of the tissues surrounding the LM during its relaxation and isometric contraction. Moreover, the greater the thinning of the ST and the thickening of the LM during its contraction, the higher the increase in lumbosacral tissue stiffness and the decrease in elasticity. Therefore, one should consider the thickness of the ST before planning or analyzing the outcomes of myotonometric or other external biomechanical measurements to avoid drawing the wrong conclusions about the mechanical properties of the myofascial tissue.

## 1 Introduction

Myotonometry is a non-invasive method capable of quantifying linear elastic and viscoelastic properties of the myofascial tissue**.** With the use of this method, greater stiffness and a decrease in low back myofascial tissue elasticity were shown in ankylosing spondylitis (White et al., 2018). Similarly, higher stiffness and tone of paravertebral muscles on the convex side in adolescents with idiopathic scoliosis (Liu et al., 2019) and higher stiffness in patients with chronic low back pain (Wu et al., 2020) have been reported. In a healthy population, myotonometry was also previously used to identify an increase in tone of the lateral abdominal wall myofascial tissue during various exercises (Park et al., 2022) and to measure changes in elasticity, tone, and stiffness of the biceps brachii and rectus femoris during different levels of voluntary contractions of these muscles (Agyapong-Badu et al., 2018). It has been indicated that myotonometry can reliably measure the stiffness of superficial myofascial tissue to a depth of 2 cm in healthy (Andonian et al., 2015) and low back pain (Hu et al., 2018) populations. However, this superficial layer contains not only the fascia and muscle tissue but also the skin that consists of the dermis and hypodermis. All these structures have different distributions and organizations of structural components, such as collagen, elastin, or glycosaminoglycans, which provide them with elasticity and tensile strength. Apart from these characteristics, there are other components such as the myofibroblasts in the fascia (Willard et al., 2012) and different muscle fiber types in the skeletal muscles (Pousson et al., 1991). In various tissues, such as the dermis (Silver et al., 2001), fascia (Yahia et al., 1993), or muscles (Taylor et al., 1997), the arrangements of the different components seem to affect tissue biomechanical properties such as stiffness and elasticity. Previously, it has been reported that skin thickness can affect the sensitivity of the instruments in detecting the stiffness of the underlying tissues (Iivarinen et al., 2014). A question to consider is how greatly morphometric proprieties of the particular superficial tissues such as the skin, fascia, or muscle, can influence myotonometric measurements performed in the lumbosacral region. Therefore, the main objective of this study was to analyze the relationships between the morphometry (thickness) and echogenicity of different layers of tissues and the mechanical properties of soft tissues in the lumbar region.

## 2 Materials and methods

### 2.1 Design

This was a cross-sectional study conducted on volunteers from the local university community. Mechanical and morphometrical properties of the lumbar soft tissues were determined at the university laboratory. The protocol of the study was granted approval from the institutional review board at the Poznan University of Medical Sciences (decision no. 709/17) and written informed consent to participate was signed by each study participant at the time of enrolment to the study.

### 2.2 Participants selection

The study included 50 adults (25 women and 25 men). The sample size was determined on the basis of previous research on the validity and reliability of myotonometry (Leonard et al., 2003) and rehabilitative ultrasound imaging (RUSI) (Koppenhaver et al., 2009). The inclusion criteria of the study were 1) age range of 20–50 years; 2) lack of low back and pelvis pain over the past 6 months; and 3) no prior lifetime history of acute low back or pelvis pain. The exclusion criteria were 1) history of severe trauma; 2) lumbar, abdominal, or pelvic surgery; 3) pregnancy within the last 12 months; 4) systemic disease; 5) skin disease in the area of measurements; 6) participation in physical training directly involving back and abdominal muscle workout within the last 3 months; and 7) a body mass index (BMI) <25 kg/m^2^.

A group was recruited from the students and staff employed at the Poznan University of Physical Education. An invitation to participate in this study was issued by means of an advertisement posted on social media and via an email sent to individual internal staff email accounts.

### 2.3 Procedure

#### 2.3.1 Preparation

Prior to measurements, participants were asked to lay in the prone position on a plinth with their head in the midline and placed in a breathing hole. One pillow was placed under the pelvis in order to minimize lumbar lordosis (Julie et al., 1992). The upper limbs were abducted in the shoulder joint to 120⁰ and flexed in the elbow joint to 90⁰. The lumbar spinous processes were palpated using the iliac crests as a reference point to determine the L4-L5 lumbar vertebral level. The myotonometry and ultrasound measurement points were marked 2 cm from the center of the L5 spinous process on both sides of the body. Ultrasound imaging was used to ensure that the measurement points were marked above the facet joint of L5/S1.

An ultrasound transducer with electroconductive gel was placed longitudinally along the spine, with the mid-point over the L5 spinous processes. Then, it was moved laterally and angled slightly vertically until the L5/S1 zygapophyseal joint could be identified. Great care was taken to not compress the skin with the transducer so as to avoid altering the subcutaneous, fascial, and muscle tissues shape.

Myotonometry and ultrasound imaging acquisition were performed during the relaxation and contraction of the lumbar multifidus muscle (LM). During relaxation, the subjects were asked to stay relaxed in the prone position. Surface electromyography (sEMG) was used to control a desired resting state of less than 5 µV (White et al., 2018). Prior to testing the contraction of the LM, all the subjects received an initial explanation about the procedure. The subjects were instructed to take a relaxed breath in and out, pause breathing, and perform contralateral arm lifting. A horizontal bar was placed 5 cm above the surface of the plinth to standardize the range of lifting. This ensured that the entire extremity was lifted.

#### 2.3.2 Data acquisition

*Myotonometry.* A MyotonPRO^®^ device (Myoton AS, Tallinn, Estonia) was used to obtain non-invasive measurements to quantify the linear elastic and viscoelastic properties of the myofascial structures at the L5/S1 level. Device-induced natural damped oscillations of the tissues were recorded with the device’s accelerometer. The measurements were taken on both sides of the spine in a randomly selected order for each participant. During each measurement, the device’s probe (3 mm in diameter) was applied vertically to the skin surface with a constant preload (0.18 N). The proper vertical position (range 90 ± 10⁰) and preloading (pressing range 3 ± 1.5 mm) of the probe were adjusted according to appropriate commands and signals provided by the device. The oscillations of the underlying tissues were evoked by delivering 10 brief (15 ms) mechanical impulses at low force (0.4 N) and 1 Hz frequency. The sampling rate was 380 Hz, and the digital acceleration sensor range was 0 ± 8 G. Three measurements during relaxation and contraction were taken at each site, and the average of the 10 impulses was used for analysis. This part of the study was conducted by an investigator with adequate experience in taking myotonometric measurements.

#### 2.3.3 Ultrasound imaging

An EnVisor C ultrasound machine (Philips, Amsterdam, Netherlands) with an L1038 linear transducer, aperture of 38 mm, and ultrasound frequency range of 5–12 MHz was used to acquire images of the LM and the associated tissues in the B-mode. The investigator and scanner were positioned to the left of the prone participant in accordance with standardized protocols used in radiology (Whittaker et al., 2007). Once a clear image was visualized, it was frozen on the screen and saved on the ultrasound scanner for later measurements. Six images during LM relaxation and contraction were taken at the level of L5/S1 for both the sides in a randomly selected order (a total of 24 images).

#### 2.3.4 Electromyography

To ensure that the LM was relaxed over the course of data collection, the sEMG measurements were performed. The electrode placement points were marked on both the sides of the body at 2 cm from the center of the L5 spinous process on the line running between the posterior superior iliac spine and the L1/L2 interspinous space. Before positioning the electrodes (22 × 28 mm Ag/AgCl self-adhesive electrodes, 20 mm center-to-center interelectrode distance), the skin was shaved, cleaned with alcohol, and abraded. The reference electrode was positioned at the radial styloid process of the right upper extremity. The sEMG signals were amplified (×1,000, SX230FW preamplifier, Biometrics Ltd., Newport, United Kingdom), band-pass filtered between 20 and 450 Hz, and transformed into digital integers (12-bit analog-to-digital conversion) at a sampling frequency of 1 kHz (DLK900, Biometrics Ltd., Newport, United Kingdom) (Kuriyama and Ito, 2005).

### 2.4 Data analysis

#### 2.4.1 Mechanical properties

The acceleration signal acquired during myotonometric measurements allows the calculation of the linear tissue elastic properties (dynamic stiffness and elasticity) and viscoelastic parameters (creep). The dynamic stiffness is expressed in newtons per meter (N/m) as the resistance of tissue to an external force that deforms its initial shape—the higher the values, the greater the stiffness. Elasticity is expressed as the logarithmic decrement of maximum acceleration between the first and second periods of signal oscillation, which is inversely proportional to the elasticity—the higher the values of decrement, the lower the elasticity (Kocur et al., 2019). The latter describes the tissue’s ability to restore its superficial shape after being deformed. Creep refers to the gradual inclination of the viscoelastic material to slowly stretch in response to the application of constant mechanical stress.

**FIGURE 1:**
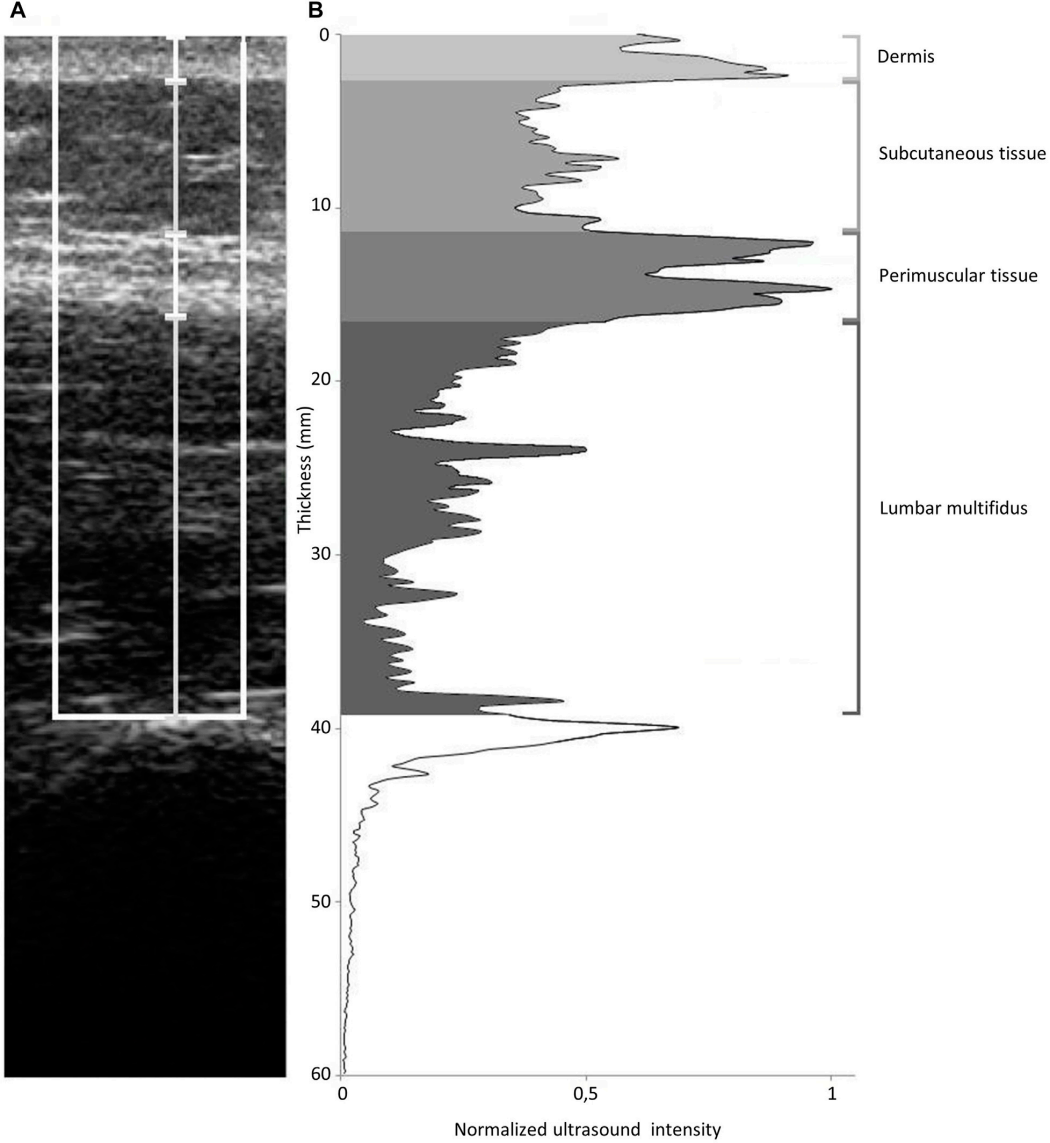
Ultrasound image analysis method. **(A)**: ultrasound image showing ROI (white box) and whisker lines corresponding to thickness of dermis, subcutaneous tissue, perimuscular tissue and lumbar multifidus. **(B)**: ultrasound intensity profile corresponding to image in A. Greyscale areas highlight the area under the curve (used as measure of echogenicity) for dermis, subcutaneous tissue, perimuscular tissue and lumbar multifidus.

*Soft tissue thickness.* Measurement of the superficial soft tissue thickness was performed according to the methodology proposed previously (Kiesel et al., 2007; Langevin et al., 2009). The LM thickness was measured from the tip of the target zygapophyseal joint to the inside edge of the superficial, hyperechoic border of the muscle (Figure 1A). The perimuscular tissue (PMT) was measured as the thickness between the hyperechoic layer from the superficial border of the PMT to the deeper border of the hypoechoic subcutaneous tissue (ST). ST thickness was measured as the distance between the cutaneous tissue and the superficial, hyperechoic border of the PMT. The dermis (D) thickness was measured between the skin surface and the border of the ST.

#### 2.4.2 Echogenicity

Echogenicity refers to the ability of reflecting or transmitting ultrasound waves (echoes) in the context of surrounding tissues. Echogenicity depends on variations in ultrasound echo reflections due in part to differences in soft tissue density. For instance, highly dense tissues such as the fibrous tissue reflect echoes brightly readily and therefore appear bright white (Stecco et al., 2019). The determination of echogenicity was performed using MATLAB software (The MathWorks, Natick, MA) according to the methodology proposed by Langevin et al. (2009). The imported ultrasound images were converted to grayscale. The region of interest (ROI) used for image data analysis was a 1-cm-wide tissue band centered on the middle of the image and located between the surface of the dermis and superior tip of the zygapophyseal joint (Figure 1B). The ROI consisted of vertical data lines. The mean gray level was calculated for each horizontal data line of the matrix describing the ROI. According to Langevin et al. (2009), the echogenicity (mm*au, arbitrary units) for individual structures was expressed as the area under the curve within the portion of the ROI outlined by the respective thickness measurements. The area under the curve was divided by the thickness of each zone to eliminate the influence of soft tissue’s zone thickness on the echogenicity, and the obtained results of echogenicity were presented as arbitrary units (au) (see the Results section).

Both soft tissue stiffness and echogenicity were acquired from the individual images of the subject’s right and left sides of the body. The thicknesses of all zones were measured during relaxation and contraction. The echogenicity was calculated only for the relaxed muscle state. The average of six measurements for each side was used for analysis.

### 2.5 Statistical analysis

The sample size was estimated *a priori* using G*Power software (version 3.1.9.2; Kiel University, Kiel, Germany) (Faul et al., 2007). In order to detect multiple linear regression at the observed coefficient of *r* ^*2*^ = 0.32 with H1 ρ^2^ = 0.30 and with 90% power (alpha = 0.05, two-tailed), G*Power suggested a total sample size of 47 participants.

The Shapiro–Wilk test was used to check the data normality distribution. Spearman’s bivariate correlation was used to assess associations between thickness and echogenicity (independent variables) and dynamic stiffness, logarithmic decrement, and creep (dependent variables) of soft tissues. Stepwise multiple linear regression with a forward selection model was conducted to test the influence of independent variables on the variance of soft tissue dynamic stiffness, logarithmic decrement, and creep. Only the independent variables that 1) showed a linear relationship with the dependent variables, 2) did not present autocorrelation (Durbin–Watson test range 1.5–2.5), 3) showed multicollinearity (variance inflation factor <2), 4) met the assumption of homodescasity, and 5) had a normal distribution of residuals were included in the model. The significance levels were determined based on *p*<0.05.

## 3 Results

The enrolled subjects’ age was 28.7 ± 7.0 years, height was 1.8 ± 0.1 m, and BMI was 22.6 ± 2.3 kg/m^2^.

### 3.1 Tissue echogenicity in relaxed state of LM

The mean values of echogenicity for different tissues layers were 0.56 au (95% CI, 0.53–0.59) for the D, 0.42 au (95% CI, 0.39–0.45) for the ST, 0.73 au (95% CI, 0.72–0.75) for the PMT, and 0.38 au (95% CI, 0.36–0.41) for the LM.

### 3.2 Relationships between myotonometric measurements and tissue echogenicity in relaxed state of LM

No correlations were found between the mechanical parameters and echogenicity of the studied tissue layers (Table 1).

**TABLE 1:** Relationships between tissues echogenicity and myotonometric measurements in lumbar multifidus relaxed state.

		D	ST	PMT	LM
Logarithmic decrement	*p*	0.212	0.591	0.131	0.085
	*r*	0.18	0.08	0.21	0.25
Dynamic stiffness (N/m)	*p*	0.797	0.230	0.058	0.940
	*r*	0.04	0.179	0.27	0.01
Creep	*p*	0.823	0.838	0.929	0.131
	*r*	0.03	0.03	0.01	0.22

D, dermis; ST, subcutaneous tissue; PMT, perimuscular tissue; LM, lumbar multifidus.

### 3.3 Relationships between mechanical properties and tissue thickness in relaxed state of LM

The greater the thickness of the ST, the lower the dynamic stiffness (very strong negative correlation) and the higher the creep (very strong positive correlation) of the soft tissues (Table 2). The thicker the PMT, the lower the dynamic stiffness (moderate negative correlation) and the higher the creep (moderate positive correlation) of the soft tissues. The results of the stepwise multiple regression analysis indicated that there was a relationship between the dynamic stiffness and ST thickness (Table 2). In the first step, the ST thickness explained approximately 37% of the variance in the dynamic stiffness. The relationship between the PMT thickness introduced in the second step and the dynamic stiffness was not significant. When the ST thickness was introduced in the first step, it explained approximately 12% of the variance in the creep. The relationships between the creep and the PMT thickness introduced in the second step were not statistically significant.

**TABLE 2:** Relationships between tissue’s zone thickness and myotonometric measurements in lumbar multifidus relaxed state.

*A) Bivariate correlation*													
				D (mm)			ST (mm)		PMT (mm)			LM (mm)	
Logarithmic decrement		*p*		0.846			0.980		0.904			0.383	
		*r*		−0.01			0.00		0.17			−0.13	
Dynamic stiffness (N/m)		*p*		0.242			<0.001		0.034			0.867	
		*r*		−0.17			−0.69		−0.30			0.02	
Creep		*p*		0.098			<0.001		0.003			0.901	
		*r*		0.24			0.79		0.41			−0.02	
*B) Regression analysis*													
	Variable		ẞ		*t*	*p*		*F*, *p*		*R* ^*2*^ add	*R* ^*2*^		Changed R^2^
Dynamic stiffness (N/m)													
Step 1	ST (mm)		−0.61		−5.13	<0.001		26.26, <0.001		0.355	0.369		0.369
Step 2	ST (mm)		−0.59		−4.01	<0.001		12.85, <0.001		0.340	0.369		0.000
	PMT (mm)		−0.01		−0.91	0.928							
Creep													
Step 1	ST (mm)		0.35		2.50	0.016		6.255, 0.016		0.103	0.122		0.122
Step 2	ST (mm)		0.46		2.62	0.012		3.666, 0.034		0.104	0.143		0.021
	PMT (mm)		−0.18		−1.03	0.307							

D, dermis; ST, subcutaneous tissue; PMT, perimuscular tissue; LM, lumbar multifidus.

### 3.4 Relationships between mechanical properties and tissue thickness in contracted state of LM

No correlations were found between the logarithmic decrement and thickness of the studied tissue layers (Table 3). At muscle contraction, the thicker the ST, the lower the dynamic stiffness (strong negative correlation) and the higher the creep (strong positive correlation) of the soft tissues. The thicker the D, the higher the dynamic stiffness (weak positive correlation) and the higher the creep (moderate positive correlation) of the soft tissues. Moreover, the thicker the PMT, the lower the dynamic stiffness (moderate negative correlation) and the higher the creep (moderate positive correlation) of the soft tissues.

**TABLE 3:** Relationships between tissue’s zone thickness and myotonometric measurements in lumbar multifidus contracted state.

*A) Bivariate correlation*												
					D (mm)		ST (mm)		PMT (mm)		LM (mm)	
Logarithmic decrement		*p*			0.558		0.816		0.400		0.239	
		*r*			0.09		0.04		0.122		0.17	
Dynamic stiffness (N/m)		*p*			0.019		<0.001		0.001		0.392	
		*r*			0.33		−0.83		−0.45		0.12	
Creep		*p*			0.003		<0.001		<0.001		0.678	
		*r*			0.41		0.85		0.48		−0.06	
*B) Regression analysis*												
	Variable		ẞ	*t*		*p*		*F*, *p*		*R* ^*2*^ add	*R* ^*2*^	Changed *R* ^*2*^
Dynamic stiffness (N/m)												
Step 1	ST (mm)		−0.73	−7.26		<0.001		52.72, <0.001		0.529	0.539	0.539
Step 2	ST (mm)		−0.69	−6.33		<0.001		26.51, <0.001		0.526	0.546	0.007
	D (mm)		−0.09	−0.82		0.415						
Step 3	ST (mm)		−0.68	−5.39		<0.001		17.40, <0.001		0.517	0.548	0.002
	D (mm)		−0.08	−0.72		0.478						
	PMT (mm)		−0.05	−0.43		0.673						
Creep												
Step 1	ST (mm)		0.85	10.6		<0.001		111.92, <0.001		0.707	0.713	0.713
Step 2	ST (mm)		0.72	8.12		<0.001		66.14, <0.001		0.739	0.750	0.037
	PMT (mm)		0.23	2.60		0.014						
Step 3	ST (mm)		0.70	7.61		<0.001		44.26, <0.001		0.738	0.755	0.005
	PMT (mm)		0.21	2.32		0.025						
	D (mm)		0.08	0.94		0.354						

D, dermis; ST, subcutaneous tissue; PMT, perimuscular tissue; LM, lumbar multifidus.

The results of the stepwise multiple regression analysis indicates that the ST thickness introduced in the first step of the regression model explained approximately 54% of the variance in the dynamic stiffness. The relationships between the dynamic stiffness and the D thickness introduced in the second step and PMT introduced in the third step were not statistically significant.

The regression analysis indicated that the ST thickness explained approximately 71% of the variance in the creep. The introduction of the PMT thickness in the second step explained approximately 4% of the variance in the creep. The relationships between the D thickness introduced in the third step were not statistically significant (Table 3).

### 3.5 Changes in myotonometric measurements and tissues thickness with LM contraction

**FIGURE 2:**
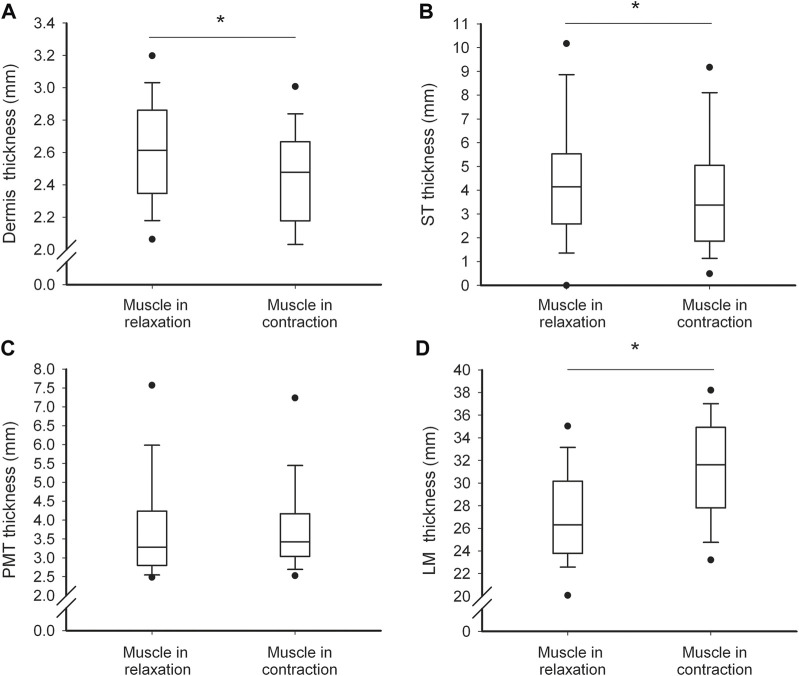
Soft tissue thickness characteristics. Values of dermis **(A)**, subcutaneous tissue **(B)**, perimuscular tissue **(C)** and lumbar multifidus **(D)** thickness in relaxed and contracted state of the muscle. Thin and thick horizontal lines within boxes denote median values, respectively. Lower and upper lines of the box indicate the 25th and 75th percentiles, respectively. Lower and upper error bars indicate the 10th and 90th percentile, respectively. Lower and upper black dots indicate 1st and 99th percentile, respectively. Asterisks indicate significant change at the level of *p*<0.05.

**FIGURE 3:**
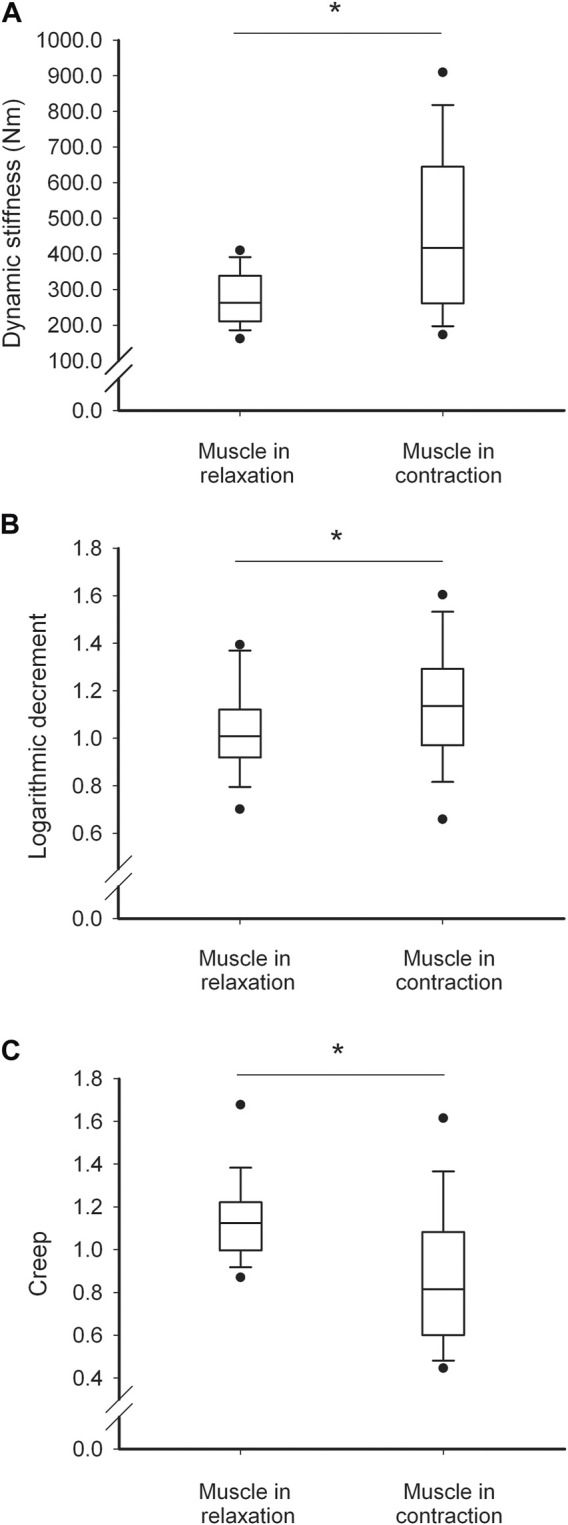
Soft tissue biomechanical parameter characteristics. Values of dynamic stiffness **(A)**, logarithmic decrement **(B)** and creep **(C)** thickness in relaxed and contracted state of the muscle. Thin and thick horizontal lines within boxes denote median values, respectively. Lower and upper lines of the box indicate the 25th and 75th percentiles, respectively. Lower and upper error bars indicate the 10th and 90th percentile, respectively. Lower and upper black dots indicate 1st and 99th percentile, respectively. Asterisks indicate significant change at the level of *p*<0.05.

With LM contraction, the tissue stiffness and logarithmic decrement (the reverse of elasticity) increased, whereas creep decreased (Figure 2). The thickness of D and ST decreased, and the thickness of LM increased, whereas the thickness of PMT did not change (Figure 3).

### 3.6 Relationships between mechanical properties and tissue thickness with LM contraction

With LM contraction, the greater the relative decrease in D thickness, the higher the increase in the dynamic stiffness (moderate negative correlation, Table 4). In addition, with the greater relative increase in the LM thickness (weak positive correlation) and decrease in ST thickness (moderate negative correlation), there was a somewhat greater relative increase in dynamic stiffness. The regression analysis indicated that the relative decrease in ST thickness introduced in the first step explained approximately 21% of the variance, and the relative increase in LM thickness introduced in the second step explained approximately 10% of the variance in the relative increase in dynamic stiffness. The relationship between the relative increase in dynamic stiffness and the relative decrease in D thickness introduced in the third step was not statistically significant (Table 4). The relationship between the relative changes in PMT thickness and logarithmic decrement was not obvious because the PMT thickness was not change with the LM contraction (Figure 2).

**TABLE 4:** Relationships between changes in relative myotonometric and tissue thickness values (expressed as % change in relation to relax state) with the transition of lumbar multifidus from relaxed to contracted state.

*A) Bivariate correlation*													
				D (%)		ST (%)		PMT (%)			LM (%)		
Logarithmic decrement (%)	*p*			0.236		0.011		0.017			0.988		
	*r*			−0.17		−0.37		0.34			0.00		
Dynamic stiffness (%)	*p*			<0.001		0.001		0.280			0.010		
	*r*			−0.51		−0.47		0.16			0.36		
Creep (%)	*p*			0.054		0.102		0.095			0.195		
	*r*			0.27		0.24		−0.24			−0.19		
*B) Regression analysis*													
		Variable	ẞ		*t*		*p*		*F, p*	*R* ^*2*^ add		*R* ^*2*^	Changed R^2^
Logarithmic decrement (%)													
Step 1		ST (%)	−0.42		−3.11		0.003		9.67, 0.003	0.159		0.177	0.177
Step 2		ST (%)	−0.40		−2.51		0.016		4.76, 0.013	0.140		0.178	0.001
		PMT (%)	0.36		0.22		0.825						
Dynamic stiffness (%)													
Step 1		ST (%)	−0.46		−3.49		0.001		12.23, 0.001	0.196		0.214	0.214
Step 2		ST (%)	−0.42		−3.35		0.002		10.14, <0.001	0.284		0.315	0.101
		LM (%)	0.32		2.56		0.014						
Step 3		ST (%)	−0.35		−2.41		0.020						
		LM (%)	0.32		2.51		0.016		7.16, 0.001	0.287		0.333	0.018
		D (%)	−0.15		−1.07		0.290						

D, dermis; ST, subcutaneous tissue; PMT, perimuscular tissue; LM, lumbar multifidus.

With LM contraction, the greater the relative decrease (thinning) of the ST, the lower the relative increase in logarithmic decrement (weak negative correlation), thus the greater the decrease in myofascial elasticity. The results of the stepwise multiple regression analysis indicate that the relative decrease in the ST thickness explained approximately 18% of the variance in the relative increase in logarithmic decrement (decrease in elasticity, Table 4).

## 4 Discussion

The aim of the study was to examine the relationships between the mechanical and morphometric properties of various superficial soft tissues surrounding the LM. The results of this study demonstrate that mechanical properties are interrelated with the thicknesses but not the echogenicity of specific tissues surrounding the LM. This was found for both the relaxed and contracted muscle states and during muscle contraction.

### 4.1 Mechanical parameters of superficial tissues at LM relaxation and contraction

In both the relaxed and contracted muscle states, dynamic stiffness and creep were related to the ST thickness. Generally, the thicker the ST, the lower the dynamic stiffness and the higher the creep in both muscle states. Moreover, in both muscle states, the PMT thickness was slightly correlated with the creep of the soft tissues, that is, the thicker the PMT, the higher the creep. The thicknesses of D and LM and the echogenicity of particular tissue layers did not correlate or negligibly correlated with the myotonometric measurements. The lack of relationship between the echogenicity and tissue mechanical properties is in agreement with previous observations that show tissue stiffness is not directly proportional to its echogenicity (Stecco et al., 2019).

The impact of skin thickness on its stiffness was documented previously (Smalls et al., 2006). The distance between the outer epidermal boundary and the inner dermal/subcutaneous fat boundary was measured, and no or negligible correlation was found between the D thickness and its stiffness. However, the layers of the D and ST were not differentiated as in the present study. Therefore, based on our results, we suppose that stiffness and creep of the superficial tissues are not related to D but to ST thickness. The ST is composed of two layers of adipose tissue: superficial and deep. Both are composed of fat lobules that can be organized in a single layer or multiple layers depending on the fat content and their thicknesses. It is suggested that ST has a great capacity for expansion and recovery from mechanical deformation; thus, it is thought to protect against external forces (Nakajima et al., 2004). Therefore, it is possible that to the same extent, the increased amount of adipose tissue contributes to decreased superficial tissue hardness and greater deformation ability, which results in lower myofascial stiffness and higher creep upon both muscle relaxation and contraction.

Although the ST thicknesses accounted for the most variance in stiffness and creep of the lumbosacral area among the studied tissue layers, a large percentage of variance in tissue mechanical parameters has been left unexplained during both muscle relaxation and contraction. This suggests that other factors contribute to the differences in stiffness, elasticity, and plasticity of the lumbosacral tissue in the prone laying position. For instance, it is unknown how the extracellular matrix morphology influences stiffness and elasticity via the content of collagen and elastin (Purslow, 2002), collagen cross-linking (Gosselin et al., 1998), and hyaluronic acid concentration (Piehl-Aulin et al., 1991). These elements compose the layers within the skin and muscle, as well as between them. The layers between the skin and muscle are organized in superficial and deep fascia. In the lower back, the superficial fascia appears as a thick fibrous layer, which fuses with the underlying muscle fascia (Markman and Barton, 1987) and is named the posterior layer of the thoracolumbar fascia (plTLF). The plTLF consists of three sublayers of parallel collagen fiber bundles and many elastic fibers with hyaluronic acid between them. Therefore, it is classified as dense regular connective tissue. Langevin et al. (2009) distinguished three types of plTLF morphology: thin, thick, and multi-layered. In our study, many subjects had the thin type. Furthermore, the epimysium, together with the perimysium and endomysium, is classified as the deep fascia of the trunk. Epimysium is a well-organized fibrous layer that ensheathes a muscle and defines its form and structure. It is composed mainly of collagen type I (Kovanen, 2002) and minor amounts of collagen type III (Riso et al., 2016), with different diameters of fibers, which are organized into three layers (Purslow, 2010). Thus, the thickness of the epimysium and hence the amount and/or diameter of collagen fibers may possibly affect the elasticity and stiffness. Therefore, rather than tissue thickness, myotonometric measurements may also be associated with many aspects of the connective tissue structure and function, which change constantly and dynamically in response to internal and external loads (Yucesoy et al., 2006). We suppose that within the PMT, not only the amount and diameter of collagen fibers but also their angle may influence the stiffness and elasticity during LM at rest and contraction. We suppose that the amount of soft tissue stiffness and elasticity may also be associated with other factors, such as collagen and elastin fibers in a proteoglycan matrix which act together and are responsible for the mechanical behavior of skin (Payne, 1991).

### 4.2 Changes in myotonometric measurements and tissues thickness with LM contraction

The superficial soft tissue mechanical parameters were unrelated to the thickness of the LM during both contraction and relaxation. We have determined that the LM layers in the zone extend approximately from 1.5 to 4.5 cm beneath the surface of the dermis. The myotonometric method can reliably measure the stiffness of the superficial myofascial tissue to a depth of 2 cm. Hence, the substantial thickness of the LM was beyond the measurement range.

Nevertheless, the transition of the LM from the relaxed to contracted state affected the thickness and mechanical parameters of the superficial tissues. We found that contraction resulted in the thickening of this muscle and thinning of the ST and dermis. This was accompanied by an increase in soft tissue dynamic stiffness and a decrease in elasticity. We have detected that, predominantly, the thinning of the ST (but not the dermis) underlaid the increase in stiffness with muscle contraction. An increase in the LM thickness with its contraction weakly defined the increase in soft tissue stiffness. Moreover, predominantly, the thinning of the ST (but not the D) underlaid the decrease in tissue elasticity with muscle contraction. We suppose that all these changes in thickness and mechanical parameters of the superficial soft tissues were partly related to the amount of LM deformation and the change in its morphometric properties with the contraction.

However, as a substantial percentage of variance has not been explained by the changes in tissue thickness, other factors presumably accounted for all the variability in the measured soft tissue dynamic stiffness and elasticity. It is known that the force produced by the contracting muscle is transmitted to the fascia in the longitudinal and transverse directions, causing anisotropic changes in the musculofascial entity (Otsuka et al., 2019). Moreover, the force of the contracted muscle is transmitted to the superficial tissues through the skin ligaments (Nash et al., 2004). Thus, perhaps with greater muscle deformation, a greater amount of force is transmitted to the surrounding superficial tissues (i.e., epimysium) (Findley et al., 2015), and greater are the changes in the soft tissue mechanical parameters. It is also possible that LM activation compresses the interstitial fluid. Perhaps under compression, the fluid is transiently trapped inside the extracellular pores formed by the endomysium and perimysium, which leads to pressure buildup (Wang et al., 2020). However, these are only hypotheses because in this study we have not assessed the amount of change in lateral force transmission to the surrounding tissues, muscle hardening, or pressure during LM contraction.

Finally, muscle contraction might change the thixotropic properties of tissues. The PMT has been shown to contain a high concentration of hyaluronic acid (Stecco et al., 2011). In the absence of mechanical loading this substance becomes more viscous, and the gliding of the fascial layers is restricted (Cowman et al., 2015). Changes in soft tissue viscosity with the transition of the muscle from a relaxed to contracted state could influence the observed changes in stiffness and elasticity.

### 4.3 Limitations

This study has to account for the following limitations. First, we did not include in our analyses a variety of other structural properties of tissues and extracellular matrix, which were probably also responsible for the variance in lumbosacral soft tissue mechanics during muscle relaxation and contraction. Second, we were unable to measure the mechanical properties of each individual superficial tissue layer. Third, we did not assess the interdependence between the biomechanical parameters and LM architecture, pennation angle, and muscle fiber fascial length. Finally, the reproducibility of the performed measurements was not checked. Thus, we are aware that we could not present a full picture of the relationships existing between the tissue’s morphological and mechanical properties.

## 5 Conclusion

In conclusion, the mechanical properties of superficial lumbosacral tissues are not associated with their echogenicity, which partly reflects differences in soft tissue density. However, we found that the thicker the subcutaneous and perimuscular layers, the lesser the stiffness and the greater the time-dependent external deformation of the tissues surrounding the LM during its relaxation or isometric contraction. Moreover, the greater the thinning of the ST and the thickening of the LM with its contraction, the greater the increase in the lumbosacral tissue stiffness and the decrease in elasticity. Thus, when performing the myotonometric measurements around the lumbosacral region or analyzing their outcomes, the ST thickness should be taken into account before any conclusions on the mechanical properties of the myofascial tissue are drawn. This can have interesting practical implications not only for researchers but also for clinicians, for example, physical therapists using manual techniques and myofascial manipulations.

## Data Availability

The raw data supporting the conclusion of this article will be made available by the authors without undue reservation.
